# Neandertal-like traits visible in the internal structure of non-supranuchal fossae of some recent *Homo sapiens*: The problem of their identification in hominins and phylogenetic implications

**DOI:** 10.1371/journal.pone.0213687

**Published:** 2019-03-12

**Authors:** Wioletta Nowaczewska, Marcin Binkowski, Anna Maria Kubicka, Janusz Piontek, Antoine Balzeau

**Affiliations:** 1 Department of Human Biology, Wrocław University, Wrocław, Poland; 2 X-ray Microtomography Lab, Department of Biomedical Computer Systems, Institute of Computer Science, Faculty of Computer and Materials Science, University of Silesia, Sosnowiec, Poland; 3 Department of Zoology, Institute of Zoology, Poznań University of Life Sciences, Poznań, Poland; 4 Department of Human Evolutionary Biology, Institute of Anthropology, Adam Mickiewicz University in Poznań, Poznań, Poland; 5 PaleoFED team «paleoanthropology: function, evolution and diversity», Departement Homme et Environnement, Museum national d'Histoire naturelle, Paris, France; 6 Department of African Zoology, Royal Museum for Central Africa, Tervuren, Belgium; University of Florence, ITALY

## Abstract

Although recently the internal structure of the non-supranuchal fossa of *Homo sapiens* has been described and compared to that observed in the Neandertal suprainiac fossa, until now it has not been examined in any modern human children. In this study, the internal structure of this fossa in the occipital bones of three children (two aged 3‒4 years and one aged 5 years ± 16 months) and one adult individual representing recent *Homo sapiens* from Australia was analysed and compared to that of the Neandertal suprainiac fossa. In order to analyse the internal composition of the fossae of the examined specimens, initially, high-resolution micro-CT datasets were obtained for their occipital bones; next, 3D topographic maps of the variation in thickness of structural layers of the occipital bones were made and 2D virtual sections in the median region of these fossae were prepared. In the fossa of one immature individual, the thinning of the diploic layer characteristic of a Neandertal suprainiac fossa was firmly diagnosed. The other Neandertal-like trait, concerning the lack of substantial thinning of the external table of the bone in the region of the fossa, was established in two individuals (one child and one adult) due to the observation of an irregular pattern of the thickness of this table in the other specimens, suggesting the presence of an inflammatory process. Our study presents, for the first time, Neandertal-like traits (but not the whole set of features that justifies the autapomorphic status of the Neandertal supraniac fossa) in the internal structure of non-supranuchal fossae of some recent *Homo sapiens*. We discuss the phylogenetic implications of the results of our analysis and stress the reasons that use of the 3D topographic mapping method is important for the correct diagnosis of Neandertal traits of the internal structure of occipital fossae.

## Introduction

The suprainiac fossa, a characteristic feature of Neandertals, was originally defined as a depression on the external surface of the occipital bone located above the inion (e.g. [1–3]). This Neandertal trait shows some degree of variation and is generally described as a transversely elongated structure, elliptical in shape, with a rough or pocked surface [4–6].

A depression above the inion has been observed in some non-Neandertal hominins, e.g. Eyasi 1 [7], Xuchang 2 [8], Manot 1 [9–10], Cioclovina 1 [11], and some representatives of recent *Homo sapiens* [4,12]. The question of the homology between these occipital depressions and those occurring in Neandertals is currently being debated (see [4,7–8,12–14]). Two categories of depressions have been identified in *Homo sapiens* specimens (fossil and recent): supranuchal and non-supranuchal fossae [4,12–14]. The supranuchal fossa has commonly been regarded as non-homologous to the Neandertal suprainiac fossa [4,12]. The non-supranuchal fossa resembles the Neandertal suprainiac fossa in size and shape and is not associated with the development of the occipital superstructures [4,12,14].

Recently, the internal composition of bone, both in the region of the suprainiac fossa in Neandertals and in that of the occurrence of the non-supranuchal fossa in *Homo sapiens*, was examined [12,13]. Computed tomography (CT) imaging (including micro-computed tomography, or μCT) data sets were used to assess the distribution of the three layers of occipital bone (external and internal tables and diploic layer) in precise median vertical and transverse virtual sections [12] and to measure total occipital bone thickness [13]. It was established that, among Neandertals, a consistent pattern of the internal structure of the suprainiac fossa was present, but one which differed from that observed for non-supranuchal fossa in *Homo sapiens* specimens. The differences between these patterns (including variations in the thicknesses of the external table and diploic layer) were interpreted by Balzeau and Rougier [12,13] as consistent with the autapomorphic character of the first trait, as suggested earlier by other authors (see e.g. [1,3,15]).

Although the aetiologies of the suprainiac fossa in Neandertals and the non-supranuchal fossa in *Homo sapiens* are still unknown, some hypotheses concerning this issue have been proposed. It has been suggested, based mainly on analysis of the morphology of the external surface of these traits, that their expression may be related to bone remodelling caused by strains influencing the crania which occur in order to retain the optimal shape of the occipital bone [4,14]. Thus these fossae have been considered convergent features indicating a common adaptive significance [14]. Balzeau and Rougier [12,13] have suggested, based mostly on the established differences in internal composition between these structures, that the functions and aetiologies of these traits can be considered significantly different. According to these authors, the limited development of the diploic layer in comparison to other occipital bone components is highly probable in the case of the development and growth of the Neandertal suprainiac fossa, and that genetic contribution can be considered the main factor influencing the expression of this trait compared with the non-supranuchal fossa in *Homo sapiens*.

The internal composition of the non-supranuchal fossa trait in *Homo sapiens* has been analysed heretofore for only three immature specimens (adolescent individuals belonging to an African Epipalaeolithic collection, specifically Taforalt XIIc2, XIIc3, and VIII), whose age at death was assessed as falling between the eruptions of the upper second and third molars; examination of these specimens was based on medical CT datasets (only the CT-2D method was used) [12]. The internal structure of the non-supranuchal fossa in *Homo sapiens* children and in immature *Homo sapiens* individuals from other regions of the world is not known, mostly because of its exceptionally rare occurrence. More studies on the non-supranuchal fossa are required in order for us to better understand the origin of the feature, its variations, and its importance for discussions of hominin evolution. In particular, more data on the internal morphology of this trait in immature *Homo sapiens* individuals are needed.

In this study we present the characteristics of the internal structure of the non-supranuchal fossa on the occipital bones of four recent *Homo sapiens* specimens derived from Australia (three children at the early stages of ontogenetic development and one adult individual) and compare them with those established by Balzeau and Rougier [12,13] for the Neandertal suprainiac fossa and the non-supranuchal fossa of *Homo sapiens*. This approach is particularly important because we can also obtain more information about variations in the depressions visible in the occipital bones of non-Neandertal hominins in the context of internal morphology, which is also related to the issue of the assessment of the origin of these fossae (e.g. in the sense of a potential inheritance from a Neandertal individual).

In this study we demonstrate the importance of detailed analysis (including the 3D mapping method) of the internal structure of the occipital bone in studies determining the presence of Neandertal traits in the internal structure of occipital depressions located above the inion point. We also discuss the significance of our results in the context of the development and aetiology of the analysed occipital depressions in comparison with the Neandertal suprainiac fossa. Finally, the results are considered in the light of the issue of the homology between the occipital fossae observed in *Homo sapiens* and those occurring in Neandertals, and of its implications for the study of human evolution.

## Materials and methods

### Sample

#### External morphology of the examined occipital bones

**Fig 1:**
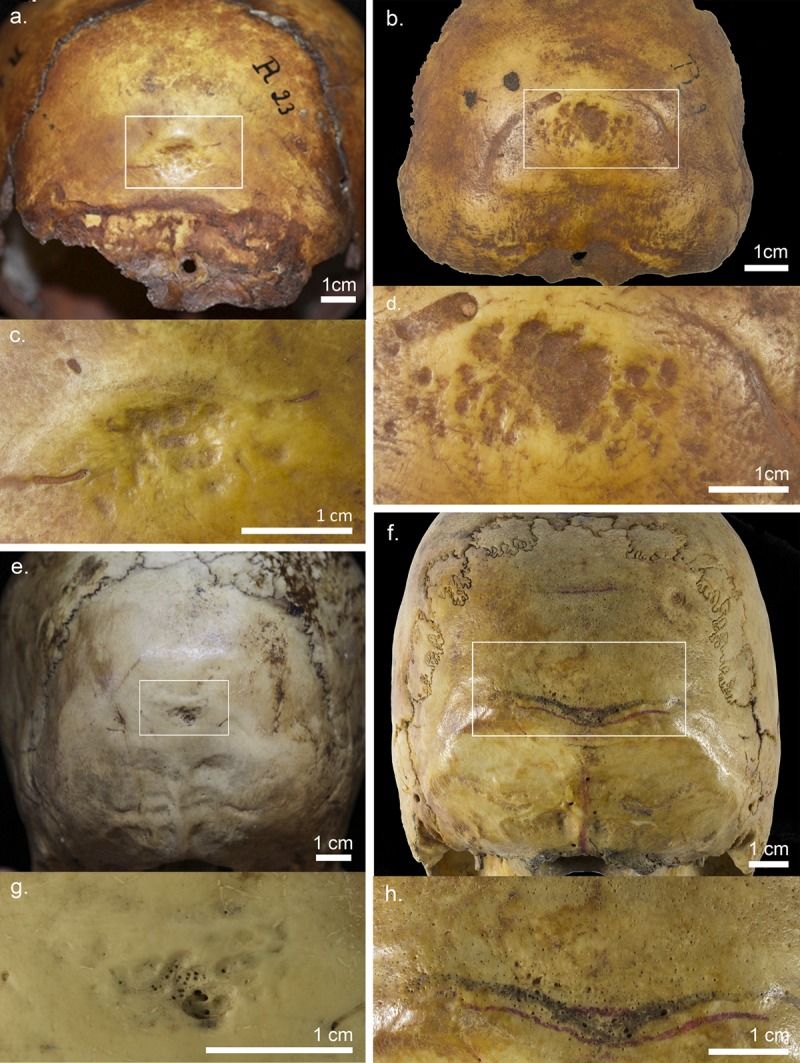
Non-supranuchal fossa visible in the occipital bones of the *Homo sapiens* specimens. The examined in this study specimens include: R23 (about 3‒4 years old child) (a, c), R24 (about 3‒4 years old child) (b, d), R82 (5 years +/‒ 16 months old child) (e, g), and R4 (adult individual) (f, h).

The cranial sample examined in this study include four recent *Homo sapiens* specimens ‒ three immature individuals (R23, R24, and R82) and one adult individual (R4)–selected because each of them exhibit a depression on the occipital bone located above the inion point which shows a resemblance in terms of external morphology to the Neandertal suprainiac fossa. All of the examined skulls belong to an Australian collection from the beginning of the twentieth century [16] stored at the Department of Human Biology, University of Wrocław (Poland). Of all (eight) crania of immature individuals in this collection, the occipital depression is visible only in the three specimens mentioned above. These depressions are characterised by an irregular surface, with small pits similar to those of the suprainiac fossa of immature Neandertals (see e.g. [4–5,17]) (Fig 1).

Based on the macroscopic examination it was found that these depressions were not caused by pathological changes resulting from bacterial infections (or other infections), artificial cranial deformation, or trepanation attempts. They differ from the lesions observed in the occipital bones of children whose heads were intentionally deformed (see e.g. [18–22]), and do not resemble changes resulting from trepanation attempts (see [23]). It is worth noting that none of the occipital bones in the examined specimens are flattened, and that the preserved cranial vaults in the examined individuals are characterised by ‘normal’ shapes (i.e. not characteristic of artificial cranial deformations; see S1–S3 Figs; see [24,25]). Once having excluded the influence of any external factor (pressure related to vault deformation) on the occipital bone in the area above the inion, it is difficult to explain the occurrence of hypothetical ‘occipital lesions’ limited to the region above the inion as being caused by bacterial infections (or other infections, e.g. those caused by head lice). It has been stressed by Holliday [20]) that neither impetigo, nor carbuncles, nor ringworm should occur exclusively on the occipital bone. Capasso et al. [26] and Capasso and Di Tota [27] have suggested that the oval area, characterised by a porous surface, that they observed above the inion in the non-deformed skulls of some recent *Homo sapiens* (part of an ancient Roman sample from Herculaneum) was probably formed in response to infections resulting from a local irritation of the periosteum related to pediculosis. It is worth emphasising that these researchers presented no detailed descriptions or images of the occipital lesions they observed in individuals of different ages at the time of death, with the exception of a single adult individual (E52) (see Fig 3, p. 128 in [26]).

In the present study, an Australian adult individual (R4) with an occipital fossa was chosen for analysis, as this individual exhibited the depression above the inion characterised by external morphology most similar to that observed in adult Neandertals, as compared with five other adult specimens in the Australian collection with non-supranuchal fossa (identified among 36 adult individuals examined by Nowaczewska [14]). The shape of the fossa of this specimen is transversely elliptical. The fossa is located above the bilaterally arched non-robust occipital torus (Fig 1F and 1H) (see also Fig 6, p. 560 in [14]). This individual was added to the analysis to establish the internal structure of this fossa and to determine whether the pattern of internal composition of the occipital depressions in the examined immature specimens is the same as that observed in this specimen.

#### State of preservation of the examined specimens

The cranial remains of the examined immature Australian individuals comprise the following: specimen R23, the vault of the cranium, including two parietal bones, the frontal bone, and a partially preserved occipital bone (S1 Fig); specimen R24, only three isolated bones (two parietal bones and the occipital bone; S2 Fig); specimen R82, a nearly complete cranium (including the facial skeleton and mandible; S3 Fig); specimen R4, a cranium missing the mandible.

#### Assessment of age at death

The age at death of individuals R23 and R24 was assessed, based on the development of the sutures and the occipital bone [28,29], at 3‒4 years; of individual R82, based on dental development [30]), at 5 years +/‒ 16 months. The cranium of specimen R4 was classified as belonging to an adult individual based on two traits: closure of the basisphenoid synchondrosis and erupted upper third molars [31].

## Methods

### Analyses of 3D topographic mapping and 2D virtual sections

**Fig 2:**
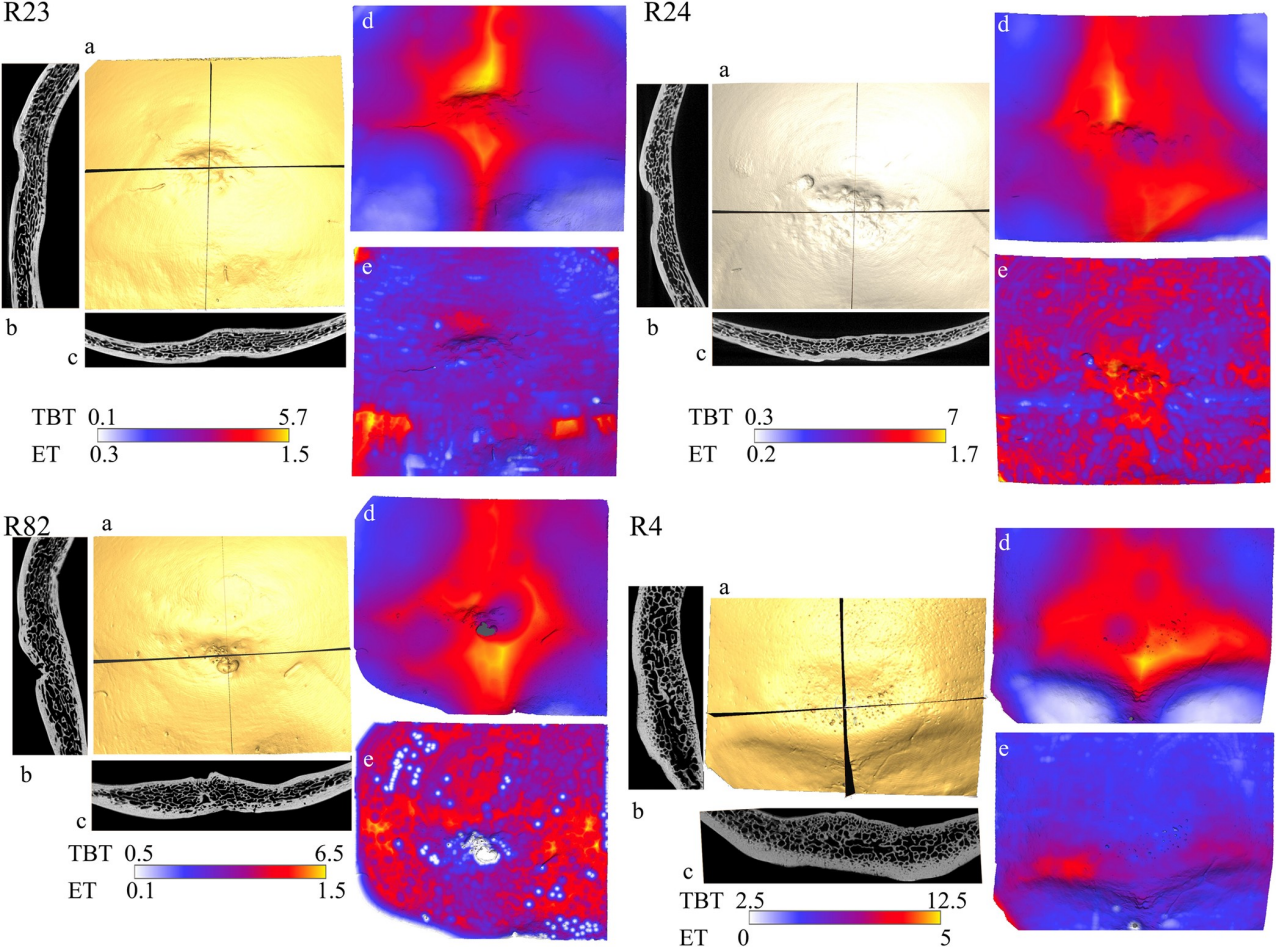
Illustration of the internal structure of the occipital bones of the examined *Homo sapiens* specimens. 3D reconstruction of the analysed area of the bone (a); vertical (b) and transversal (c) slices along the largest extension of the occipital depression; 3D mapping of total bone thickness (d) and of the external table (e); and minimal and maximal values for total bone thickness (TBT) and external table thickness (ET) as boundaries of the chromatic scale used to display each chromatic map. The presented scales are in centimeters.

The occipital bones of the examined individuals were imaged using X-ray micro-CT (v|tome|x s, GE Sensing & Inspection Technologies, phoenix|x-ray, Wunstorf, Germany). High-resolution imaging datasets were obtained for all specimens, with resolutions of 36.6 μm for R23, R24, and R82 and 50.09 μm for R4. Data from the imaging methodologies were used to obtain information about the structural composition of the occipital bones in the area, including the region of the fossa (see [12,13]). Three-dimensional (3D) topographic mapping, concerning variations in the thicknesses of whole bone and external table, was obtained from the segmented data and presented using a chromatic scale, from the lowest (white) to the highest (yellow) values of this trait (Fig 2). Based on these datasets for each specimen, virtual sections (sagittal and transverse) of the occipital bones in the median region of the fossa were created according to the methodology used by Balzeau and Rougier [12] (Fig 2).

In each specimen’s dataset, the borders between the external table and diploic layer as well as with the surrounding air were established by manual segmentation (SMM: Seuillage Manuel Multiple, or Multiple Manual Thresholding; [32]). This procedure is based on the measurement of the median value (or half maximum height, HMH) of the CT values of the two components that define the analysed interface [33,34]. Manual segmentation must be used each time the attenuation coefficient of one of the components varies along the interface. This enables the precise identification of the interface between the two components despite local fluctuations in CT numbers [32]. This segmentation protocol ensures accurate isolation of the bone area in order to quantify the exact extent of the cranial vault and outer table. No overflow artefacts were present in the examined datasets. A specific protocol was used to ensure accurate isolation of the various components of the images. Micro-CT data were needed to conduct a precise analysis of the inner structure of the occipital bone, given the potential limitations of this study concerning the spatial resolution of the datasets and the partial-volume-averaging artefact (which is related to imaging methodologies) [35]. Avizo 7 (Mercury Computer Systems) was used to conduct the following tasks: multiplanar reformatting, thresholding procedures, three-dimensional volume rendering, 3D topographic mapping (with the Surface-Distance module), and acquisition of illustrations. Although the 3D-mapping method overcomes some limitations concerning the use of slice-based imaging methodologies (2D) to analyse the thickness of bone layers, the latter method was also used in our study (see [12, 36]). All examined specimens are accessible by others in a permanent repository of Department of Human Biology (University of Wroclaw, Poland). The sample used in this study include the following numbers of the specimens: A-0R4, A-0R23, A-0R24 and A-0R82.

### Criteria for evaluating the similarity of the internal structure of the examined occipital fossae to Neandertal suprainiac fossae

Taking into account the main aim of the present study, which concerned the assessment of the similarity of the internal structure of the examined occipital fossae to Neandertal suprainiac fossae, two criteria were established, based on the results obtained by Balzeau and Rougier [12,13]. The first criterion concerns variation in the thickness of the external table (ET) of the occipital bone. In Neandertals, no substantial thinning of the ET was observed in the region of the suprainiac fossa, as opposed to the supranuchal and non-supranuchal fossae in *Homo sapiens* (see [13]). No substantial decrease was observed in the thickness of the ET (the outer and inner borders of the ET run parallel to one another) in a median vertical section/slice of the region of Neandertal suprainiac fossa occurrence. Thus, when we observe a lack of substantial thinning in the ET in the area of the occipital fossa as well as in relation to the regions of the occipital bone surrounding it, we can identify this trait as Neandertal-like.

The second criterion concerns variation in the thickness of total bone (TB) and of the diploic layer of the occipital bone. In Neandertals, regions of thinning of TB in the area of the suprainiac fossa (as well as relative to the regions of the surrounding occipital bone without superstructures) were visible [13]. No substantial thinning of TB in the area of the occipital fossa was observed in *Homo sapiens* individuals examined by Balzeau and Rougier [13]. The thinning of the diploic layer in the region of the Neandertal suprainiac fossa was also identified in the median vertical section of the latter (where the presence of the internal occipital crest was assumed) in relation to the area located above it (see [13]). Thus, when we observe regions of substantial thinning of TB in the area of the fossa co-occurring with the thinning of the diploic layer, we can identify this trait as Neandertal-like.

It should be explained that in the present study the term ‘Neandertal-like’ concerns solely an observed similarity (as defined above) to Neandertals in the analysed traits of internal structure of occipital fossae, without suggesting a Neandertal origin for these traits.

## Results

### The R23 specimen

An oval depression with a pocked surface (within an area approximately 1.0 × 1.5 cm) is located in the medial part of the occipital squama, in the lower part of the convex occipital plane of the R23 specimen (Fig 1A and 1C). This depression is asymmetrical in shape; its surface exhibits small pocked depressions of various sizes located to the left and right. The upper limit of the large depression is convex. The depression causes an invagination in the bone which is deeper at its superior than at its inferior extent.

Concerning variations in external table (ET) thickness (Fig 2E–R23), the maximal values of this trait were observed in the area of superior nuchal lines (mainly the area including the lateral parts of these lines): yellow and red regions, < 0.5 cm; small red and pink region above the upper border of the depression, < 0.4 cm. There is no substantial decrease in outer table thickness between the region of the depression and the areas on both sides of this structure, which is slightly more pronounced when compared to the relief located above the depression. The round blue spots correspond to areas of slight reduction in ET thickness, related to the occurrence of small pockets in the region of the fossa and of the pocked surface that extends bilaterally (Fig 2E–R23). The parallel courses of the outer and inner borders of the ET can be observed in the vertical and transverse sections of the fossa region in the R23 specimen (see Fig 2B and 2C–R23). In accordance with the first criterion used in this study concerning the internal structure of the Neandertal suprainiac fossa, the above-mentioned traits can be considered Neandertal-like. The general variation in the shape of the ET related to the presence of the depression is visible. This shows that some bone reaction occurred in response to an event occurring exocranially, causing some remodelling of the internal extension of the ET. As a result, thinning of the ET can be observed at the sites of external pocking of the surface of the bone (see Fig 2E—R23).

Regarding variations in total bone (TB) thickness, the maximal values identified in the central part of the occipital plane (yellow and red regions, < 0.7 cm) are related to the internal occipital protuberance and the occurrence of internal occipital crests (Fig 2D—R23). The highest values of thickness correspond to the presence of the internal crests: regions marked in yellow are visible above and below the area of the fossa (Fig 2D—R23). The total thickness of the bone in the region where the fossa is present decreases in relation to the areas of the bone above and below this structure (above and below the red area of the fossa, yellow areas of the bone are visible; above and below the pink area of the fossa, red areas of the bone are visible); the thinning of the diploic layer can be observed in a 2D median vertical section of this fossa (Fig 2B—R23). The co-occurrence of the thinning of TB and of the diploic layer is similar to that observed in Neandertal suprainiac fossae. It is worth emphasising that, in some areas of the fossa (not related to the presence of the internal occipital crest–the right part of this depression) (see Fig 2D—R23), the thinning of TB in the regions of the cupules is visible in relation to the adjacent regions of the fossa. This observation may indicate thinning of all three layers of the bone in these cupules.

### The R24 specimen

A large asymmetric depression, triangular in shape, is observed in the centre of the occipital bone of the R24 specimen. Pocked areas are visible bilaterally. This alteration of the external surface extends over an area approximately 2.6 × 0.9 cm (Fig 1B and 1D). A small ridge runs along the uppermost extension of the depression (Fig 1B and 1D).

Concerning the variations in the thickness of the ET of the occipital bone, the maximal values are identified in the area of the depression (except in the presence of cupules) and the area surrounding this structure (yellow and red regions) as well as in other parts of the occipital squama (red regions) (Fig 2E—R24). This pattern corresponds to an inflammatory process and reaction of the bone. Inflammation caused some resorption of bone thickness, but also some redeposition of the ET as part of the healing process [37]. This explains the relative thickening of the ET as well as its irregular distribution alongside and all around the depression. It is worth emphasising that the use of the 3D mapping method (as opposed to the 2D method) enables identification of the above-mentioned changes indicating the occurrence of the inflammatory process in the occipital bone of the R24 specimen. Although the parallel course of the outer and inner borders of the ET is observed in the median vertical section of the occipital fossa region, the data presented above concerning the irregularity in the thickness of the ET clearly indicate that the ET of the occipital fossa of this specimen does not exhibit a Neandertal-like structure.

The pattern of variations in TB thickness in the R24 specimen is generally similar to that occurring in R23 (Fig 2D—R24). The total thickness of bone in some areas of the region of the occipital depression (its central and right parts, i.e. the pink region) is less that above and below this structure (red and orange regions, < 0.7 cm) (Fig 2D—R24), indicating the thinning of the diploic layer. The thinning of this layer is also clearly visible in the median vertical section of this fossa (2D analysis; see Fig 2B—R24). According to the second criterion of the internal structural similarity to the Neandertal suprainiac fossa used in this study, this trait could be considered Neandertal-like; however, in the case of this specimen, the influence of pathological changes on the formation of this trait is certain.

### The R82 specimen

A deep depression, elongated in shape and occupying an area of approximately 0.8 × 1.5 cm, is visible in the centre of the occipital bone of the R82 specimen. Around and above this depression, other small depressions, varying in size, shape, and depth, are visible (Fig 1E and 1G). Small perforations can be observed over the entire surface of the occipital bone and are especially numerous in the area of the fossa. In some regions of this depression, the bone is very thin (Fig 1E and 1G).

In terms of variations in ET thickness, the maximal values are identified in the lateral areas of occipital bone on the left and right sides of the depression and in the area above it (yellow and orange areas, < 0.5 cm) (see Fig 2E—R82). Thickness is greatly reduced throughout the depression. In some locations the diploic layer nearly communicates with the exocranial surface due to remodelling of the bone (the external boundary of the diploic layer is affected locally). In the median vertical section of the depression of the R82 specimen (see Fig 2B—R82), perforations in the ET are observed, along with a reduction in its thickness; these traits are also visible in the transverse section of this depression (Fig 2C—R82). The concentrations of white spots (related to the presence of the perforations) similar to those observed in the occipital depression are also visible in other regions of the bone (e.g. in the view of the ET of the occipital bone, in the right lower corner; see Fig 2E—R82). The co-occurrence of these perforations and the thinning of the ET indicate the presence of pathological changes. Thus no Neandertal-like traits are observed in the ET structure of the R82 specimen.

In terms of variations in TB thickness, the maximal values are identified in the central area of the occipital squama above and below the depression (yellow and red regions, < 0.5 cm) (Fig 2D—R82). The depression corresponds to marked but irregular thinning of the bone. In this specimen, the thinning of the whole bone mentioned above was most probably caused by pathological changes. It is important to stress that in the R82 specimen a specific concentration of porosity is observed in the greater wing of the sphenoid bone (visible in norma lateralis of the skull) and on the medial side of the coronoid processes of the mandible (see S4A and S4B Fig). Orbital lesions (cribra orbitalia) are also present in this specimen (S4C and S4D Fig). These observations may indicate a significant deficiency of vitamin C in this individual, and thus the occurrence of scurvy [38–40]. Nor can we exclude the possible presence of anaemia in this individual (based on the identification of cribra cranii and cribra orbitalia) [41–45]. In Neandertals, thinning of the whole bone in the region of the fossa, caused by the thinning of the diploic layer, is observed; in the R82 specimen, the thinning of the bone in the corresponding region is mostly related to the thinning of the external table resulting from pathological changes. Although slight thinning of the diploic layer (in the region of the fossa) is observed in the median vertical section (2D analysis) (Fig 2B—R82), in this specimen no Neandertal-like traits were identified in the internal structure of the occipital fossa.

### The R4 specimen

The well-preserved occipital bone of the R4 specimen exhibits a depression above the inion (Fig 1F and 1H), exhibiting an external morphology which, among those identified by Nowaczewska [14] in other adult *Homo sapiens* crania belonging to the same cranial collection, is the most similar to the Neandertal suprainiac fossa. This depression is visible above the torus occipitalis and depresses the upper limit of this superstructure, causing its apparently arched shape in this specimen. The shape of the occipital fossa is transversely elliptical and occupies an area approximately 3.1 × 1.5 cm (see Fig 1F and 1H).

In terms of variations in ET thickness, the maximal values are identified in the area of the torus occipitalis (red area, < 0.5 cm, visible on the right part of this superstructure) (Fig 2E–R4). There is no substantial difference in thickness between the area of the depression and the regions of the occipital plane located above it. These values are lower than those of the areas located below the depression, illustrating a clear thinning of the ET here in relation to areas of the occurrence of the superstructure. This pattern is clearly visible in the median vertical section of the occipital depression of the R4 specimen (see Fig 2B—R4). The same condition, with clear thinning of the ET in the area of the depression relative to lateral areas (the places where the superstructure is present), can be seen on the transverse plane (Fig 2E—R4). Taking into account that no pathological changes were observed in the ET or TB of the R4 individual (using macroscopic examination and the 3D mapping method), and in light of the first criterion of similarity to the Neandertal suprainiac fossa used in this study, the lack of substantial thinning of ET in the region of occipital depression observed in this specimen in relation to the areas of the occipital bone without superstructures can be considered a Neandertal-like trait. However, the lateral variations show that the external table was affected by the presence of the depression.

In terms of variations in TB thickness, the maximal values are identified in the central area of the occipital squama, including the region of the torus occipitalis, the greater part of the area of the depression, and immediately above it (yellow and red areas, < 1.2 cm) (Fig 2D—R4). The location of maximal total bone thickness is related to the superstructure (torus occipitalis) and internal occipital protuberance with internal occipital crests. The depression is characterised by a slight reduction in total bone thickness. No thinning of the diploic layer is observed in the region of the fossa in its median vertical and transverse sections (Fig 2B and 2C—R4). Thus no Neandertal-like traits are visible in the variation in TB thickness of the occipital fossa of the R4 specimen.

In summary, the results indicate the presence of Neandertal-like traits in the internal structure of the occipital fossa in two of the four specimens examined in this study. These traits are visible in the pattern of variation in ET and TB thickness of the fossa of the R23 specimen. One of these traits (concerning ET) was identified in the fossa of the R4 specimen, another (concerning TB) in that of the R24 specimen. However, in the R24 specimen, the similarity in this trait can be apparent (probably related to the influence of a pathological process on the internal structure of the occipital bone). No Neandertal-like traits were observed in the internal structure of the fossa of the R82 specimen, in which the presence of pathological changes which influenced that structure was identified.

## Discussion

The occipital depressions observed in three examined immature specimens of recent *Homo sapiens* (R23, R24, and R82) do not co-occur with either strongly developed highest or superior nuchal lines or with the external occipital protuberance; thus they cannot be considered examples of supranuchal fossae. These superstructures are not completely formed at the early stages of ontogenetic development of the human occipital bone, and thus the presence of occipital depressions in these specimens cannot be considered to be related to their specific formation. These depressions were classified as the second type of occipital fossae identified to date in *Homo sapiens*, i.e. non-supranuchal fossae (see [12–14]). The occipital depression of adult specimen R4 was also classified as this type of fossa, based on its significant similarity in terms of external morphology to the suprainiac fossae of adult Neandertals (see also [14]). However, its shape is related to an arrangement of occipital superstructures which differs from that of Neandertals. It is also important to note that the size and shape of the area of the fossae examined in this study in immature specimens differ from those in Neandertal children (which exhibit larger and more elliptically-shaped suprainiac fossae) (for comparison see e.g. a Neandertal child—La Ferrassie 8; Fig 2, p. 41 in [13]). Additionally, in the R23 specimen we can observe a small elevation of the bone above the fossa, which is not characteristic of Neandertals.

In making assumptions regarding the development and aetiology of the occipital fossae examined here, their internal structure should be also considered. In this study, the presence of Neandertal-like trait(s) in the internal structure of the occipital fossa was established in two individuals of recent *Homo sapiens*, one immature (R23), one adult (R4). In these specimens (as opposed to R24 and R82) no pathological changes can be considered to have caused the formation of the Neandertal-like traits of the internal structure of their occipital depressions. In the R24 specimen the areas of the resorption of the external surface of the occipital bone (the external table of the bone was affected by the osteoclasts’ over-activation–contrary to hemorrhagic processes), together with the areas of the new bone deposition, indicate the occurrence of the inflammatory process. These changes are also visible in images of the virtual sections of this bone [37,46,47]. This process was probably caused by local post-traumatic necrosis or an epidermal cyst (see [37]). The presence of the advanced anemia, scurvy, or hemangioma were excluded because the pattern of the internal structure of the R24 occipital bone was different from that characteristic of these diseases (see [47]). It is worth emphasising that no Neandertal-like traits have been diagnosed in the internal structure of the non-supranuchal fossae of recent *Homo sapiens* examined to date (see [12–13]). Thus, the results of the present study show that the variability in their internal morphology is greater than was previously thought. Use of the 2D method alone (based mainly on interpretation of the view of the median vertical section of the area of occipital depression) excludes the potential certain identification of pathological processes reflected in changes in the pattern of variability of ET thickness of the occipital bone, which in turn influences the internal structure of the examined fossae. In this study, this has been demonstrated using the example of two individuals, R24 and R82. The 3D mapping method enables analysis of the ‘whole picture’ of the occipital fossa, e.g. it enables comparison of the data obtained for the median part of the fossa with those established for its lateral parts. This is important because of differences between individuals in the location of the internal occipital crest and/or the eminentia cruciata relative to the area of the occipital depression.

### The importance of the location of the eminentia cruciata in the occipital bone

Different organisation of the exo- and endocranial surfaces of the occipital bone are observed in *Homo sapiens* and Neandertals. In Neandertals (both adult and immature individuals) the endinion (defined as “Crossing point of the four legs of the eminentia cruciata”—[48]:Table 2, p. 492) is located below the inion (defined as: “Intersection of the midsagittal plane with the tangent connecting the most superior point of the superior nuchal line” ([48]: Table 2, p. 492). The area of the Neandertal suprainiac fossa corresponds to the inferior part of the sagittal sulcus (visible as an internal occipital crest in the transverse section of this fossa) on the internal surface of the occipital bone, thus no influence of the eminentia cruciata on the thickness of the diploic layer in the area of this fossa can be observed. In *Homo sapiens* the endinion may be located either below (most frequently) or above the inion [48]. The 3D model of R4 shows that endinion is located above inion and coincides with the upper extent of the occipital depression. In the image of the median vertical section of the occipital depression (Fig 2B—R4) we can see an elevation of the internal surface of the occipital bone indicating the presence of the eminentia cruciata and thus an increase in the thickness of the diploic layer in this location. In the case of the immature *Homo sapiens* specimens examined in this study, the endinion is located below the position of the occipital depression in R24 (based on direct evaluation) and within the region of the occipital depression in R23 (based on direct evaluation); however, additionally, based on the location of the internal occipital crest, which is visible in an image of the transverse section (Fig 2C—R23), we can state that the endinion is located (in relation to the occipital fossa) on the left side, not in the central part, of this depression. This means that the eminentia cruciata, as an elevation of the inferior surface of the occipital bone, cannot be seen on an image of the median vertical section of this fossa; in the lower part of the fossa in R82 (based on the analysis of the 3D mapping image of the thickness of the whole occipital bone–note the location of the right leg of the eminentia cruciata marked in red and pink in Fig 2D—R82), similarly to the R23 specimen, the internal occipital crest is located in the left part of the area of the fossa; accordingly, in this case as well, the elevation related to the eminentia cruciata cannot be seen on an image of its median vertical section (Fig 2B—R23). The examples presented above clearly show that use of the 3D mapping method precludes potentially incorrect interpretations of images of the median vertical sections of occipital depressions (e.g. related to an asymmetric location for the internal occipital crest). Thus, to be certain of the validity of interpretations of the internal structure of the occipital depression occurring in hominins, the 3D mapping method should be used. This suggestion applies particularly to examples of hominins in which the presence of Neandertal traits in the occipital depression is suggested based solely on 2D analysis (e.g. Xuchang 2 [8]).

### The issue of the development and aetiology of non-supranuchal fossae with Neandertal-like traits in their internal structure

The suprainiac fossa has been identified in the early stages of Neandertal ontogenetic development, e.g. it is visible in the occipital bones of two Dederiyeh infants (1 and 2) whose age at death was assessed at less than 2 years [49–50] and in all immature Neandertal individuals (see e.g. [17, 51–54]. Balzeau and Rougier [12,13] observed no non-supranuchal fossae in the youngest individuals in the African and European samples of *Homo sapiens* included in their study; accordingly, they suggested that the distribution of non-supranuchal fossae in relation to age is different in *Homo sapiens* than in Neandertals. However, an occipital depression with a markedly pocked surface may be present early in the development of some recent *Homo sapiens*; such a depression is visible on the occipital bone of a child aged three years (see Fig 7, p. 233 in [4]) and in individuals examined in this study aged 3‒4 (R23 and 24) and 5 years (R82). As in Neandertal children and juveniles [1,5,17, 52–55], these occipital depressions show more pocked external surfaces than are found in an adult individual (R4). The results of our study indicate that, among the non-supranuchal fossae identified in *Homo sapiens*, two subtypes of these depressions can be distinguished: first, without Neandertal-like traits visible in their internal structure (see [12,13]), and second, with these traits. The development of the first of these subtypes followed a different pattern from that of Neandertals, as suggested mainly by substantial remodelling of the outer edge of the ET of the occipital bone in the region of the fossa (the outer and inner borders of the ET do not run parallel to each other) which has not been identified in Neandertals (see [12,13]). It has been suggested that the function of this subtype, as opposed to the Neandertal suprainiac fossa, is related to maintenance of the optimal shape of the occipital bone [13].

In accounting for the occurrence of Neandertal-like traits in the second subtype of the non-supranuchal fossa in the examined *Homo sapiens* specimens, it is important to consider whether these traits could have developed in the same way suggested for Neandertals. Although current knowledge on the development and cause of the Neandertal suprainiac fossa is far from complete, some assumptions have been made concerning this issue. According to Balzeau and Rougier [13], the substantial reduction of the whole bone at the site of the occurrence of cupules observed in the suprainiac fossa of immature Neandertals may have resulted from the processes of modality of growth and development of the occipital bone, not from external factors. In the case of the R23 specimen we can observe two types of cupules. Most are related solely to substantial reduction of the thickness of the external table of the bone, but some are formed by reductions in total bone thickness (these are visible in the right part of the area of the fossa; see Fig 2E—R23). The first type of cupule can suggest the influence of external factors on their formation, e.g. related to strains generated by muscles; the second type probably developed similarly to those in Neandertals, but we cannot assess this unequivocally.

According to Balzeau and Rougier [13], the occipital depression in Neandertals resulted not from resorption of the external surface of the bone, but most probably from the limited development of the diploic layer in relation to other regions of the occipital bone. Although, in the case of the occipital depression in the R23 specimen, the diploic layer is thinner than in other areas of the bone, it is difficult to assess whether the origin of this trait is the same as in Neandertals. Neither do we know whether this trait might be present in this specimen as it is in the adult individual. It is worth mentioning that the diploic venous system was identified in the area of a Neandertal child’s suprainiac fossa (see La Ferrassie 8), as opposed to adult Neandertal specimens, but its role in the expression of the above-mentioned trait is unknown [13]). Thus additional studies are necessary to resolve this issue, including other Neandertal individuals and also *Homo sapiens* specimens in various stages of ontogenetic development. The main difference between the occipital fossa in specimen R23 and a Neandertal child’s suprainiac fossa is the thickness of the whole bone in the region of this structure. In Neandertal children (as opposed to *Homo sapiens*) it is very thin, for reasons unknown; thus the answer to this question could be a helpful clue in the solution of the issue presented above.

Taking into account the observations that, in examined adult individual R4, the diploic layer is relatively thick in the region of the fossa, and that no cupules are observed on the external surface of this depression, we can suppose that, during the growth and development of the occipital bone of this individual, the thickness of the diploic layer increased to a more substantial extent than in Neandertals. Some remodelling of the external surface of the external table in the region of this depression may have occurred, as has also been suggested in the case of Neandertals (see [13]).

Although the assumption that the second subtype of the non-supranuchal fossa in adult *Homo sapiens* is a retention of the juvenile fossa and that it is strongly genetically determined (without functional significance) may be the simplest interpretation, it is more likely to apply to the Neandertal suprainiac fossa because of its common occurrence compared to *Homo sapiens* [13]. The size and shape of the region of the occipital depression observed in immature Neandertals is similar to that in adults. It is worth mentioning that some adult recent *Homo sapiens* specimens examined by Nowaczewska [14] exhibit non-supranuchal fossae which are generally similar in terms of the size and shape of their area of occurrence to those observed in the immature specimens examined in this study (see Fig 4a, b, p. 558 in [14]). Thus, further research is necessary, including analyses of their internal structures as well as those of other *Homo sapiens* individuals exhibiting similar external morphology of the occipital depression.

It has been stressed by Caspari [4] that studies of the development of the occipital bone in mammals indicate that the appearance of the fossa above the inion may be related to the developmental process which leads to the correct formation of the cranial vault. At the current stage of our knowledge (including the results of this study), the interpretation mentioned above is the most probable cause of the second subtype of formation of non-supranuchal fossae in the occipital bone of some *Homo sapiens* children; however, further studies are needed. This suggests an adaptive function (in the sense described above) for this subtype of non-supranuchal fossae (see [4]). It can be also assumed (based on the traits of the fossa of the R4 adult individual) that their expression in adult individuals may be additionally modified, but it is difficult to establish the cause of this process.

The observed morphological differences between the examined occipital fossae of *Homo sapiens* (external in the case of R23 and R4; internal in the case of R4) and Neandertal suprainiac fossae may suggest that the development of the former differed from that of the latter, and, as is worth emphasising, probably differed as well from that suggested by Balzeau and Rougier [13] for non-supranuchal fossae of *Homo sapiens* without Neandertal-like traits in their internal structure (unrelated to the substantial resorption of ET thickness in the area of the fossae). In light of these interpretations, along with the suggested aetiology of the examined fossae, which differs from that assumed for Neandertals, they cannot be considered homologous to Neandertal suprainiac fossae. However, further studies on this issue are needed.

### Phylogenetic implications

The results of this study support the interpretation of the Neandertal suprainiac fossa as a Neandertal autapomorphy. However, they make it much more difficult to answer the question of how similar the external and internal morphology of the occipital depression occurring in non-Neandertal hominins should be to Neandertal suprainiac fossae in order to consider it as being inherited from Neandertals. This is an important issue, because recent genetic studies have indicated a DNA exchange between Neandertals and Palaeolithic *Homo sapiens* as well as between them and Denisovans (whose cranial morphology is currently unknown) (see [56]). Additionally, in the literature, examples of possible hybrids between the above-mentioned types of hominins have been proposed (e.g. the cranial remains Cioclovina 1, Oase 2, and Muierii 1, thought of as belonging to *Homo sapiens* but exhibiting some Neandertal traits, including occipital fossae; see [11,57]). However, to date the internal structure of their occipital depressions is unknown and the assumption of their Neandertal origin is based only on external morphology.

In light of the results obtained in this study, we can consider the following hypothesis: the variant of the occipital fossa established here may be an example of the morphological variability of the occipital depression above the inion occurring in representatives of Middle/Late Pleistocene hominins. The identification of the same variant of this depression in recent sub-Saharan African *Homo sapiens* in which Neandertal DNA is not present (which can be assumed probable, based on observations of the absence of Neandertal DNA in the DNA of recent sub-Saharan African *Homo sapiens* populations; see [53]) may support this interpretation (excluding the possible influence of Neandertal genes on the appearance of Neandertal-like traits in the internal structure of these fossae). Although a small percentage of Neandertal DNA has recently been identified in Amhara (in Ethiopia) and Toubou (in Chad) populations, this has been interpreted as the result of gene flow from Eurasian *Homo sapiens* carrying Neandertal DNA to *Homo sapiens* from Central and East Africa [58]. To test the hypothesis mentioned above, further studies are needed, including more recent *Homo sapiens* with non-supranuchal fossa from different regions of the world, especially Africa. According to this hypothesis it is considered probable, as was proposed earlier by Trinkaus [7], that Middle Pleistocene populations of hominins that may have been ancestors of *Homo sapiens* or early representatives of our species may exhibit more variation in the morphology of the suprainiac region than has been assumed in the case of recent *Homo sapiens*. Thus it is important to obtain detailed data about the internal structure of the non-supranuchal occipital fossa, whose occurrence has been diagnosed in hominins, e.g. the African Eyasi 1 [7] (dated to ~ 200‒400 ka) and Aduma [59] (dated to about ~ 79‒105 ka) hominins (see [10]). For example, it has been indicated that the calvaria of Manot 1, a *Homo sapiens* specimen from Israel dated to 54.7 ± 5.5 kya, exhibits an occipital fossa involving only the external table of occipital bone, which therefore is not homologous to the Neandertal suprainiac fossa [9–10] and which, moreover, is different from the second subtype of the non-supranuchal fossa described in this study. It is worth emphasising that one of the largest Middle Pleistocene samples of hominins derived from one site, namely Sima de los Huesos (Atapuerca in Spain, dated to 430 ka), considered as belonging to the Neandertal clade exhibiting occipital depressions, requires an analysis of internal morphology, as this may provide exceptional information about the morphological variability of these depressions [60,61].

Recently the early Late Pleistocene cranium of Xuchang 2, an adult hominin from Asia (China, dated to 105,000‒125,000 years ago), was described as exhibiting an occipital depression, indicating some Neandertal traits in external and internal morphology, and suggesting the influence of Neandertal genes on its occurrence [8]. Although this interpretation is most parsimonious, in light of the previously mentioned hypothesis it is also possible that the occipital depression in the Xuchang 2 specimen could constitute ‘retention’ of the ‘archaic version’ of this fossa which occurred in populations of its Middle Pleistocene ancestors prior to the appearance of hominins with fully Neandertal morphology. It is worth mentioning that the occipital depression of Xuchang 2 is most similar in external morphology to that of the immature *Homo sapiens* specimen R23 and also to one occurring in a recent African *Homo sapiens* specimen examined by Nowaczewska (see Fig 4a, p. 558 in [14]). However, to test the hypothesis mentioned above, more data resulting from the use of 3D topography mapping should be obtained and used to conduct a precise comparison between the internal structure of the occipital depressions of fossil hominins and of recent *Homo sapiens*.

## Supporting information

S1 Fig The preserved parts of the cranium of specimen R23. View of the cranium in: norma lateralis (a); norma verticalis (b).
(TIF)

S2 Fig The preserved parts of the cranium of specimen R24. The view of the two parietal bones and the occipital bone.
(TIF)

S3 Fig The cranium of specimen R82. View of the cranium in norma lateralis (a); in norma basalis (b); mandible with preserved teeth (c).
(TIF)

S4 Fig The cranium of specimen R82. Abnormal porosity visible on the greater wing of the sphenoid bone (a), on the medial surface of the coronoid process of the mandible (b), both probably related to the presence of scurvy; cribra orbitalia visible in the orbital vault (c and d).
(TIF)
