# Supplementary figures and images for: Neandertal-like traits visible in the internal structure of non-supranuchal fossae of some recent Homo sapiens: The problem of their identification in hominins and phylogenetic implications
Source: PLoS One. 2019 Mar 12;14(3):e0213687. doi: 10.1371/journal.pone.0213687 (PMC6421632; doi:10.1371/journal.pone.0213687)

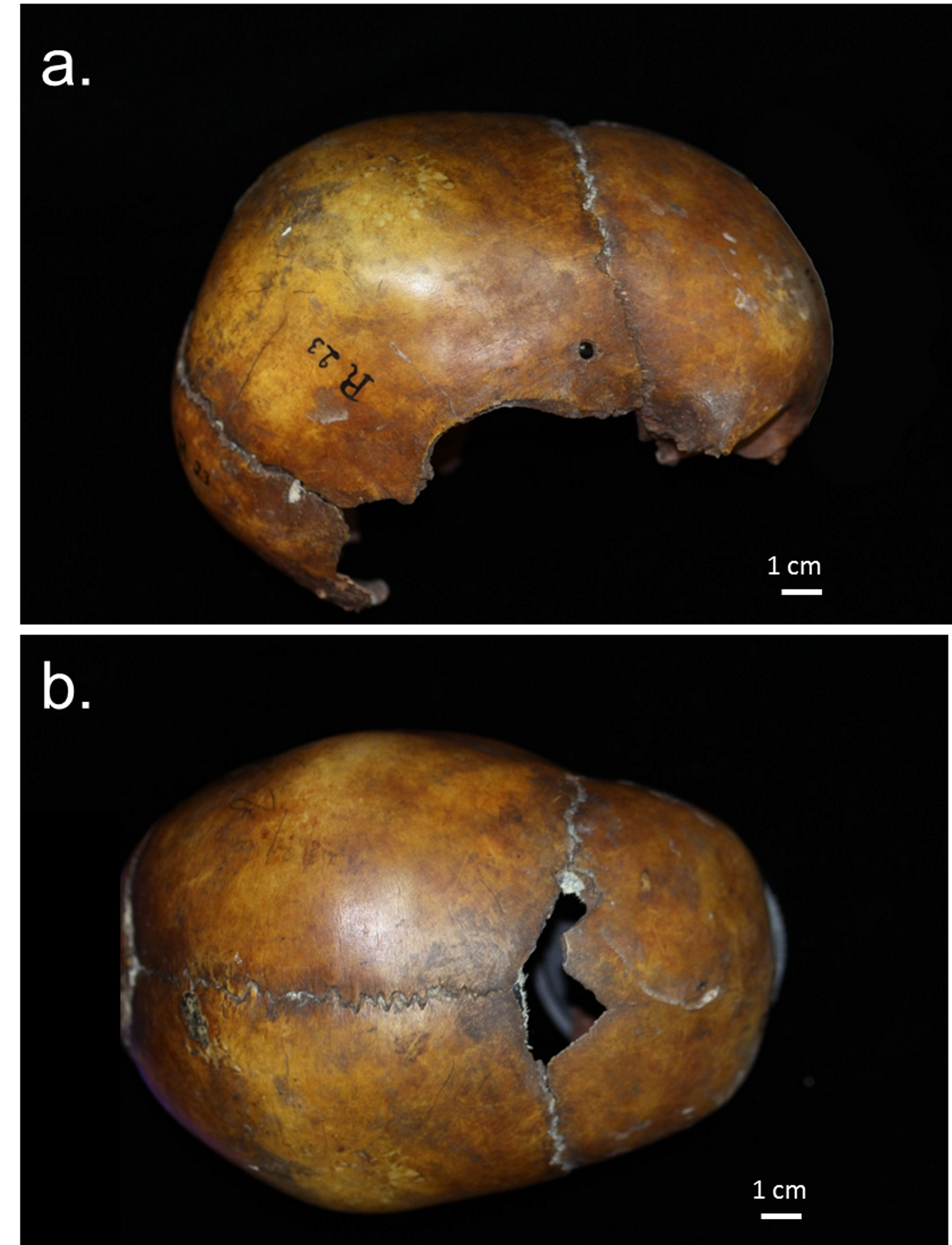

Supplement: S1 Fig — View of the cranium in: norma lateralis (a); norma verticalis (b). (TIF) [file pone.0213687.s001.tif]

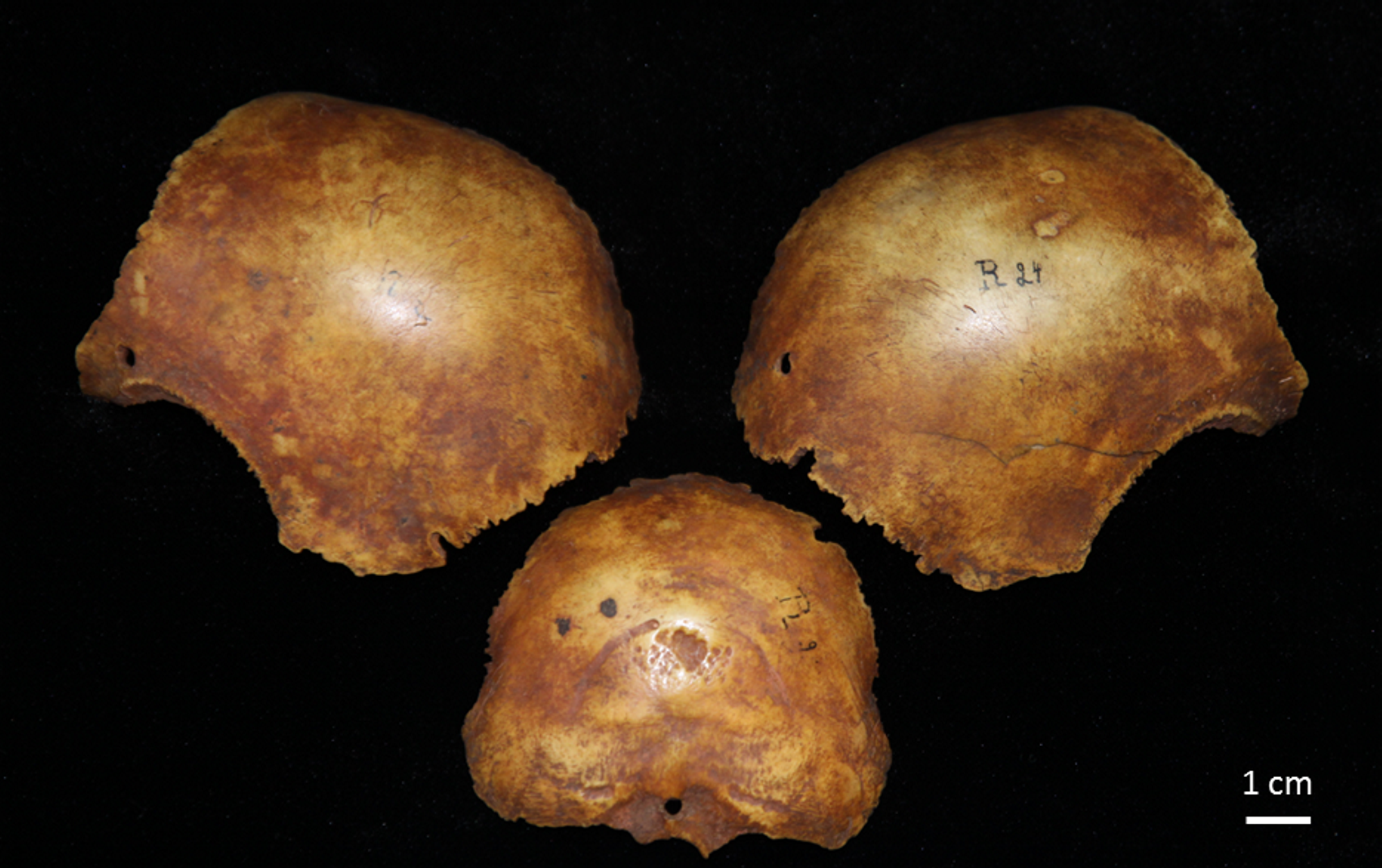

Supplement: S2 Fig — The view of the two parietal bones and the occipital bone. (TIF) [file pone.0213687.s002.tif]

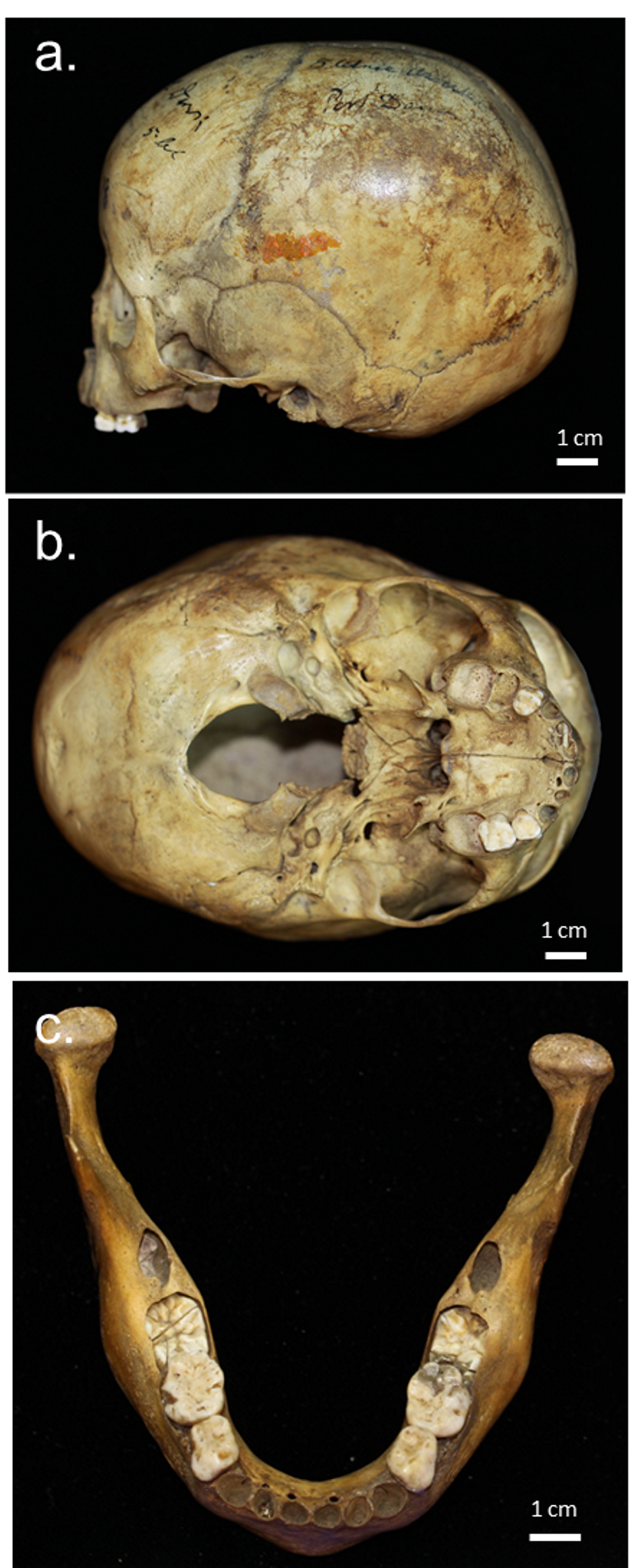

Supplement: S3 Fig — View of the cranium in norma lateralis (a); in norma basalis (b); mandible with preserved teeth (c). (TIF) [file pone.0213687.s003.tif]

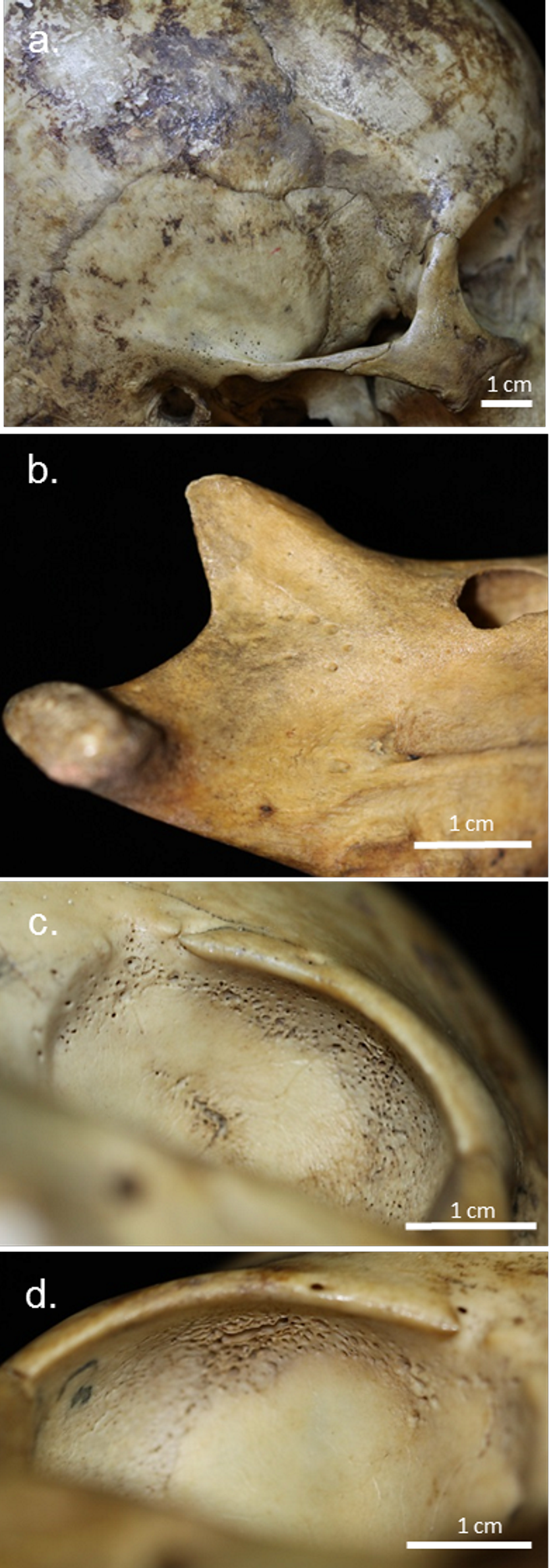

Supplement: S4 Fig — Abnormal porosity visible on the greater wing of the sphenoid bone (a), on the medial surface of the coronoid process of the mandible (b), both probably related to the presence of scurvy; cribra orbitalia visible in the orbital vault (c and d). (TIF) [file pone.0213687.s004.tif]
